# CRASH-3 - tranexamic acid for the treatment of significant traumatic brain injury: study protocol for an international randomized, double-blind, placebo-controlled trial

**DOI:** 10.1186/1745-6215-13-87

**Published:** 2012-06-21

**Authors:** Yashbir Dewan, Edward O Komolafe, Jorge H Mejía-Mantilla, Pablo Perel, Ian Roberts, Haleema Shakur

**Affiliations:** 1 Department of Neurosciences, FLT. Lt. Rajan Dhall Fortis Hospital, Vasant Kunj, Delhi, NCR New Delhi, 110070, India; 2 Department of Surgery, Obafemi Awolowo University Teaching Hospital, Ile-Ife, Osun State, 20022, Nigeria; 3 Department of Anaesthesia and Resuscitation Fundación Valle del Lili, Carrera 98#18-49, Cali, Colombia; 4 Clinical Trials Unit, London School of Hygiene & Tropical Medicine, Keppel Street, London, WC1E 7HT, UK

**Keywords:** Antifibrinolytic, Clinical trial, Emergency care, Intracranial bleeding, Tranexamic acid, Traumatic brain injury

## Abstract

**Background:**

Worldwide, over 10 million people are killed or hospitalized because of traumatic brain injury each year. About 90% of deaths occur in low- and middle-income countries. The condition mostly affects young adults, and many experience long lasting or permanent disability. The social and economic burden is considerable. Tranexamic acid (TXA) is commonly given to surgical patients to reduce bleeding and the need for blood transfusion. It has been shown to reduce the number of patients receiving a blood transfusion by about a third, reduces the volume of blood transfused by about one unit, and halves the need for further surgery to control bleeding in elective surgical patients.

**Methods/design:**

The CRASH-3 trial is an international, multicenter, pragmatic, randomized, double-blind, placebo-controlled trial to quantify the effects of the early administration of TXA on death and disability in patients with traumatic brain injury. Ten thousand adult patients who fulfil the eligibility criteria will be randomized to receive TXA or placebo. Adults with traumatic brain injury, who are within 8 h of injury and have any intracranial bleeding on computerized tomography (CT scan) or Glasgow Coma Score (GCS) of 12 or less can be included if the responsible doctor is substantially uncertain as to whether or not to use TXA in this patient. Patients with significant extracranial bleeding will be excluded since there is evidence that TXA improves outcome in these patients. Treatment will entail a 1 g loading dose followed by a 1 g maintenance dose over 8 h.

The main analyses will be on an ‘intention-to-treat’ basis, irrespective of whether the allocated treatment was received. Results will be presented as appropriate effect estimates with a measure of precision (95% confidence intervals). Subgroup analyses for the primary outcome will be based on time from injury to randomization, the severity of the injury, location of the bleeding, and baseline risk. Interaction tests will be used to test whether the effect of treatment differs across these subgroups. A study with 10,000 patients will have approximately 90% power to detect a 15% relative reduction from 20% to 17% in all-cause mortality.

**Trial registration:**

Current Controlled Trials ISRCTN15088122; Clinicaltrials.gov NCT01402882

## Background

Worldwide, over 10 million people are killed or hospitalized because of traumatic brain injury (TBI) each year [1]. Approximately 90% of deaths from TBI occur in low- and middle-income countries [2]. TBI predominantly affects young adults, and many patients experience long lasting or permanent disability. The social and economic burden of TBI is considerable. With rapidly increasing motorization, the incidence of TBI is predicted to rise in low- and middle-income countries [3]. An effective, widely practicable and affordable treatment for TBI could save many thousands of lives and substantially reduce the burden of disability.

The antifibrinolytic agent tranexamic acid (TXA) is commonly given to surgical patients to reduce bleeding and the need for blood transfusion. A systematic review of randomized trials of TXA in elective surgical patients shows that TXA reduces the number of patients receiving a blood transfusion by about one-third, reduces the volume of blood transfused by about one unit, and halves the need for further surgery to control bleeding [4]. These differences are all highly statistically significant. Furthermore, there is no evidence of any increased risk of vascular occlusive events with TXA [4].

More recently, TXA has been shown to reduce mortality in trauma patients with significant extracranial bleeding. The CRASH-2 trial, which enrolled 20,211 bleeding trauma patients from hospitals in 40 countries, showed that the administration of TXA within 8 h of injury significantly reduces deaths due to bleeding ((RR Relative Risk) = 0.85, 95% confidence interval (CI) 0.76 to 0.96; *P* = 0.008), and all-cause mortality (RR = 0.91, 95% CI 0.85 to 0.97; *P* = 0.0035) compared to placebo, with no apparent increase in vascular occlusive events [5]. Among patients treated very soon after injury, the reduction in mortality with TXA is even greater [6]. Cost-effectiveness analysis has shown that the administration of TXA to bleeding trauma patients is highly cost effective in low-, middle- and high-income settings [7]. As a consequence of the CRASH-2 trial results, TXA has been incorporated into trauma treatment protocols worldwide and has been included on the World Health Organization (WHO) List of Essential Medicines.

The knowledge that TXA reduces blood loss in surgery and reduces mortality in traumatic bleeding raises the possibility that it might also be effective in TBI. Intracranial hemorrhage is common after TBI, and is associated with increased mortality and disability. In the Medical Research Council (MRC) CRASH-1 trial, which included 10,008 TBI patients, 73% of patients with moderate or severe TBI had intracranial hemorrhage on computerized tomography (CT) scan [8]. Hemorrhage size is strongly associated with outcome. Patients with a large intracranial hemorrhage, whatever the location, have a substantially higher mortality than patients with a small hemorrhage [9]. In many TBI patients, the intracranial bleeding continues after hospital admission [10,11]. Among patients with moderate or severe TBI, who are found to have intracranial bleeding on a CT scan taken soon after hospital admission, intracranial bleeding progresses in 84% of patients.

Approximately one-third of patients with TBI have laboratory evidence of abnormal coagulation at hospital admission [12]. These patients have an increased risk of intracranial hemorrhage and higher mortality. Increased fibrinolysis, as indicated by high levels of fibrinogen degradation products, is common in TBI and is a strong independent predictor of progressive intracranial hemorrhage [13]. These observations raise the possibility that TXA might reduce intracranial hemorrhage and improve outcome in TBI patients.

In addition, it has been shown that progressive tissue damage and edema develops in regions surrounding intracranial bleeding lesions, and is associated with a worse outcome [14]. Tissue plasminogen activator (tPA) has been shown to be an important factor in this process of peri-lesional edema [15-17]. By blocking the conversion from plasminogen to plasmin, TXA counteracts the effect of tPA and, therefore, it is possible that TXA might also be beneficial in traumatic intracerebral hemorrhage by decreasing peri-lesional edema through a specific neuroprotective effect.

Two studies have evaluated the effect of TXA in traumatic brain injury. The CRASH-2 Intracranial Bleeding Study was a nested, randomized trial conducted in 270 trauma patients who had evidence of TBI on a pre-randomization CT scan. A second scan was conducted 24 to 48 h after randomization. There was a reduction in intracranial hemorrhage growth (RR = 0.80; 95% CI 0.59 to 1.09), fewer ischemic lesions and lower all-cause mortality (RR = 0.60; 95% CI 0.32 to 1.11) in TXA allocated patients, but these results were not statistically significant [18]. A second randomized trial conducted in 240 patients with isolated TBI also found reductions in hemorrhage growth (RR = 0.56; 95% CI 0.32 to 0.97) and mortality (RR = 0.67; 95% CI 0.34 to 1.32) with TXA, but this trial did not collect data on ischemic lesions [19]. Meta-analysis of the two trials shows a significant reduction in hemorrhage growth (RR = 0.72; 95% CI 0.55 to 0.94) and mortality (RR = 0.63; 95% CI 0.40 to 0.99) with TXA.

Although the results from these trials are promising, the estimates are imprecise and there are no data on the effect of TXA on disability. Furthermore, because patients in the CRASH-2 Intracranial Bleeding Study also had significant extracranial bleeding, the extent to which the results can be generalized to patients with isolated TBI is open to question. The CRASH-3 trial will provide reliable evidence about the effect of TXA on mortality and disability in patients with TBI. The effect of TXA on the risk of vascular occlusive events and seizures will also be assessed. If such a simple and widely practicable treatment was shown to improve outcomes in patients with TBI, then it could be used in high-, middle- and low-income countries, saving many thousands of lives and reducing the burden of disability.

### Objective

The CRASH-3 trial will provide reliable evidence as to whether the antifibrinolytic agent TXA can reduce mortality and disability in patients with traumatic brain injury.

## Methods, design, discussion

### Overview

**Figure 1:**
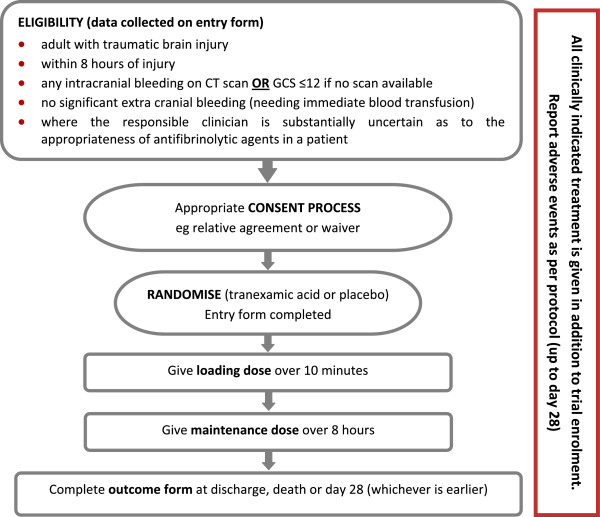
Trial Overview.

The CRASH-3 trial is an international, multicenter, pragmatic, randomized, double-blind, placebo-controlled trial to quantify the effects of the early administration (within 8 h of injury) of TXA on death and disability in TBI patients. A total of 10,000 adult TBI patients who fulfil the eligibility criteria will be randomized to receive either TXA or placebo. The eligibility criteria are based on the uncertainty principle (Figure 1).

#### Pragmatic design and the uncertainty principle

The pragmatic design will allow us to find out how effective the treatment actually is in routine everyday practice. Ethically, this randomized controlled trial can only be undertaken if there is collective scientific uncertainty about which of the interventions being compared is more likely to benefit patients [20,21]. However, for an individual clinician to be able to recommend enrolment of a patient into a trial, they must be substantially uncertain about the appropriateness of the trial treatment in that particular patient. The eligibility criteria for the CRASH-3 trial are based on this uncertainty principle. This approach to assessing trial eligibility is well established [22].

A patient can be enrolled if, and only if, the responsible clinician is substantially uncertain as to which of the trial treatments would be most appropriate for that particular patient. A patient should not be enrolled if the responsible clinician or the patient (or his/her representative) are for any medical or non-medical reasons reasonably certain that one of the treatments that might be allocated would be inappropriate for this particular individual (in comparison with either no treatment or some other treatment that could be offered to the patient in or outside the trial). Using the uncertainty principle should allow the process of this trial to be closer to what is appropriate in normal medical practice.

#### Eligible patients

Adults with TBI who are within 8 h of injury, with any intracranial bleeding on CT scan, or, if no scan is available, who have a Glasgow Coma Score (GCS) of 12 or less, and no significant extracranial bleeding (that is, not in need of immediate blood transfusion) are eligible, if the responsible clinician is substantially uncertain as to the appropriateness of TXA for them. The fundamental eligibility criterion is the responsible clinician’s ‘uncertainty’ as to whether or not to use TXA in a particular patient with TBI. This pragmatic approach will allow us to see whether the intervention improves patient outcomes under real-life conditions.

Although some increase in the risk of vascular occlusive events (arterial or venous thrombosis) might be expected with TXA on theoretical grounds, clinical trials in trauma patients have not found any increase [4-6]. In the CRASH-2 trial, in which 20,211 trauma patients were randomly assigned within 8 h of injury to either TXA (loading dose 1 g over 10 min followed by infusion of 1 g over 8 h) or placebo, there were fewer vascular occlusive events in patients allocated to receive TXA (168 (1.7%) TXA versus 201 (2.1%) placebo; RR = 0.84; 95% CI 0.68 to 1.02).

Because TXA is eliminated by renal excretion there is a risk of accumulation in patients with renal impairment. However, because the CRASH-3 trial involves a very short course of TXA (a loading dose followed by an infusion over 8 h) the risk of accumulation should be minimal.

Although high doses of TXA have been associated with seizures in patients undergoing cardiac surgery, there were no reports of serious unexpected adverse events involving seizures in the 20,211 trauma patients randomized into the CRASH-2 trial, half of whom received the dose of TXA that is being used in the CRASH-3 trial [23].

Even though there are no absolute contraindications to TXA administration in patients with traumatic brain injury, patients with TBI should only be enrolled if their doctor is reasonably ‘uncertain’ as to whether or not to use TXA for that particular patient. The summary of product characteristics for TXA and an Investigator’s Brochure will be provided to investigators to ensure that they have the information needed to assess the balance of harms and benefits in each patient.

#### Randomization

Patients will receive all-usual treatment for TBI. Patients eligible for inclusion should be randomized and the study treatment started as soon as possible. The Entry Form (Additional file 1: Form 1) will be used to assess eligibility and collect baseline information. The next consecutively numbered treatment pack, taken from a box of eight packs, should then be chosen. Once a patient has been randomized, outcome data need to be collected even if the trial treatment is interrupted or is not actually given.

#### Follow-up

No extra tests are required for the trial but an Outcome Form (Additional file 2: Form 2) should be completed 28 days after randomization, or at death or hospital discharge if either happens sooner. Short-term disability will be assessed on the Outcome Form using the Disability Rating Scale (DRS). This scale measures the level of disability in six diagnostic categories of (1) eye opening, (2) best verbal response, (3) best motor response, (4) self-care ability for feeding, grooming and toileting, (5) level of cognitive functioning, and (6) employability, and can be used across the span of recovery. The maximum score a patient can obtain is 29, which represents an extreme vegetative state. A person without disability would score zero [24].

We will also assess specific patient orientated outcomes that have been identified by patients and their families as being important. They were identified from the literature and then considered and agreed by patient representatives from RoadPeace, the UK national charity for those killed or injured in road crashes.

#### Settings

Patients will be recruited from hospitals in high-, middle- and low-income countries. There is no limit to the maximum number of patients to be recruited at each site.

#### Number of patients needed

Two main factors determine the number of patients needed in a trial: the estimated event rate and size of the treatment effect. The primary end point for CRASH-3 is death in hospital within 28 days.

#### Estimated event rate

In the CRASH-1 trial, among patients with moderate or severe TBI (GCS of 12 or less), the risk of death in the control group was approximately 20%.

#### Sample size and size of treatment effect that should be detectable

A study with 10,000 TBI patients would have about 90% power (two sided alpha = 1%) to detect a 15% relative reduction (from 20% to 17%) in all-cause mortality. With 10,000 patients, the study would also have over 90% power to detect a difference in mean DRS score of 1.0 (assuming a SD of DRS of 9.0). Experience from the CRASH-1 and CRASH-2 trials suggests that the anticipated rates of loss to follow-up (less than 1%) would not impact importantly on study power.

#### Recruitment of collaborating investigators

The trial will recruit hospitals from many countries around the world and we will continue to add hospitals throughout the trial until the sample size is achieved. Suitable collaborating hospitals and investigators will be assessed in terms of the trauma service that they provide and their ability to conduct the trial. Before the trial can begin at any site, the local Principal Investigator must agree to adhere to Good Clinical Practice Guidelines and all relevant national regulations. In addition, all relevant regulatory and ethics approvals should be in place before the trial starts at a site.

#### Ethical considerations, information giving and written informed consent

The Glasgow Coma Score (GCS) is a method of assessing the level of consciousness in TBI patients. Patients with a GCS score of 15 are generally considered fully conscious, but those with a GCS score of 12 or less are not fully conscious and would not be mentally capable of giving informed consent to participation in a clinical trial. Intracranial bleeding is a clinical sign indicating significant brain injury, and patients with this diagnosis would not be physically or mentally capable of giving informed consent to participation in a clinical trial. Therefore, given that patients are eligible for inclusion in the CRASH-3 trial if they have sustained a traumatic brain injury and have either intracranial bleeding on a CT scan or a GCS of 12 or less, they will, by default, be physically or mentally incapable of giving consent.

Traumatic brain injury is an emergency condition that requires urgent treatment. Because intracranial bleeding occurs soon after injury, any treatment needs to be given as soon as possible. There is evidence from trials in traumatic extracranial bleeding that TXA is more effective when given early [25]. The need for urgent treatment in the CRASH-3 trial means that the implementation of the research cannot be delayed and that it would be inappropriate to delay treatment until fully informed consent can be obtained from a relative or other legal representative. Patients who are incapable of giving consent in emergency situations are an established exception to the general rule of informed consent in clinical trials. This is clearly acknowledged in the Declaration of Helsinki.

“Research involving subjects who are physically or mentally incapable of giving consent, for example, unconscious patients, may be done only if the physical or mental condition that prevents giving informed consent is a necessary characteristic of the research population. In such circumstances the physician should seek informed consent from the legally authorized representative. If no such representative is available if the research cannot be delayed, the study may proceed without informed consent provided that the specific reasons for involving subjects with a condition that renders them unable to give informed consent have been stated in the research protocol and the study has been approved by a research ethics committee. Consent to remain in the research should be obtained as soon as possible from the subject or a legally authorized representative.” (WMA Declaration of Helsinki 2008 – Ethical Principles for Medical Research Involving Human Subjects)

**Figure 2:**
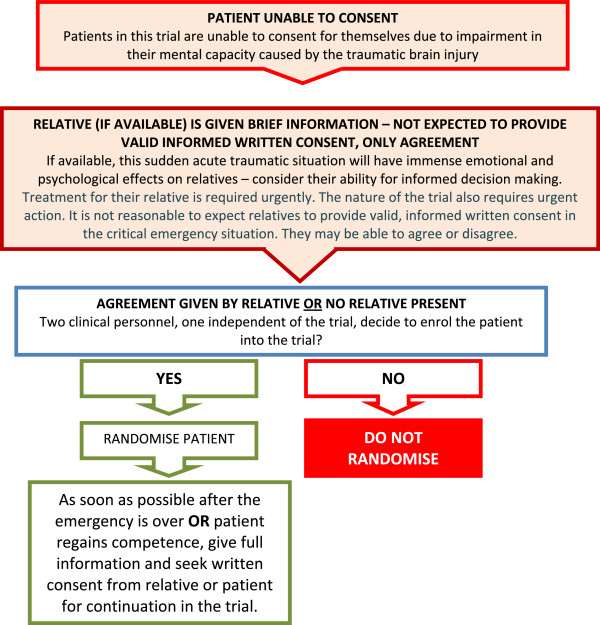
Consent procedure diagram.

The following procedure which is in accordance with the Declaration of Helsinki will be used for giving information and obtaining informed consent for the CRASH-3 trial (Figure 2).

#### Prior information giving

If relatives are present, bearing in mind the clinical situation and their level of distress, they will be provided with brief information about the trial. Specifically, the responsible doctor will explain to the relatives that the patient will receive the usual emergency treatments for traumatic brain injury but that, in addition to these, the patient has been enrolled in a research study that aims to improve the treatment of patients with this condition. It will be explained that the study is being done to see whether using a drug called TXA will help patients with head injury by reducing the amount of bleeding into the brain, therefore preventing further brain damage. The relative will be informed that the patient will be given an infusion into a vein over 8 h of either TXA or a dummy medicine (a liquid which does not contain TXA). The doctor will explain that TXA has been shown to improve outcome in patients with other types of severe injury and that, whilst we hope that it will also improve recovery after head injury, at present we cannot be sure about this. Further information will only be provided on request. If requested, a brief information sheet will be provided (Additional file 3: Form 3). If relatives object to the inclusion of the patient in the trial, their views will be respected. If no relatives are present, two doctors (one independent of the trial) will consider the patient’s eligibility criteria and any known views of the patient about trial participation. Together they will decide whether or not to enrol the patient into the trial. Examples of the information sheets and consent forms to be used are given in the additional files provided (Additional file 4 and 5).

### Randomization

Randomization codes will be generated and secured by an independent statistical consultant from Sealed Envelope Ltd (London, UK). The codes will be made available to a GMP certified clinical trial supply company explicitly for the treatment packs to be created in accordance with the randomization list. Eligibility will be determined from the routinely collected clinical information and no trial specific tests are required. Patients eligible for inclusion should be randomized to receive either TXA or placebo (sodium chloride 0.9%) and the trial treatment started as soon as possible.

Baseline information will be collected on the Entry Form (Additional file 1: Form 1) and the next lowest consecutively numbered pack will be taken from a box of eight treatment packs. When the treatment ampoules are confirmed as being intact, the patient is considered to be randomized into the trial. The entry form data will be sent to the Trial Coordinating Centre as soon as possible after entry. Once a patient has been randomized, the outcome of the patient should be obtained even if the trial treatment is interrupted or is not actually given.

### Treatment

TXA will be compared with matching placebo (sodium chloride 0.9%).

### Dose selection

TXA has been used to reduce bleeding in elective surgery for many years. A systematic review of randomized trials of TXA in surgery shows that dose regimens of TXA vary widely [4]. Loading doses range from 2.5 mg/kg to 100 mg/kg and maintenance doses from 0.25 mg/kg/h to 4 mg/kg/h delivered over periods of 1 to 12 h. Studies examining the impact of different doses of TXA on bleeding and transfusion requirements showed no significant difference between a high dose and a low dose [4,26].

In emergency situations, the administration of a fixed dose is more practicable because weighing patients in such situations is difficult. In the CRASH-3 trial, a fixed dose of 1 g loading dose of TXA, followed by a 1 g maintenance dose over 8 h has been selected. This fixed dose is within the dose range which has been shown to inhibit fibrinolysis and provide hemostatic benefit. It should be efficacious for larger patients (>100 kg) but also safe in smaller patients (<50 kg), as the estimated dose/kg that the latter group would receive has been used in other trials without adverse effects. Furthermore, this fixed dose was used in 20,211 patients enrolled in the CRASH-2 trial and was found to be both effective and safe. The same fixed dose was also used in two studies of TXA in TBI patients, again with no evidence of adverse effects.

### Drug manufacture, blinding and supply of trial treatment

The active trial drug TXA (Cyklokapron® Injection) will be purchased on the open market. TXA is manufactured by Pfizer Ltd under Marketing Authorization Number PL00032/0314. The Marketing Authorization guarantees that the product has been manufactured and released in accordance with the UK Good Manufacturing Regulations.

Placebo (sodium chloride 0.9%) will be manufactured specially to match the TXA by a GMP certified manufacturer.

Ampoules and packaging will be identical in appearance. The blinding process and first stage Qualified Person (QP) release will be done by the designated clinical trial supply company. The blinding process will involve complete removal of the original manufacturer’s label and replacement with the clinical trial label bearing the randomization number which will be used as the pack identification. Other pack label text will be identical for both TXA and placebo treatments and will be in compliance with requirements for investigational medicinal products.

The designated clinical trial supply company will also be responsible for maintaining the Product Specification File (PSF) until final database lock and unblinding of the trial data. Quality control checks to assure the blinding process will be performed on a random sample of final QP released drug packs. High-performance liquid chromatography (HPLC) analyses, separation of known TXA, will be assessed against blinded samples to confirm which ampoule contains the placebo and active treatments. The tested samples will be unblinded to assure accuracy of blinding.

The Trial Coordinating Centre (TCC) will be responsible for assuring all relevant approvals are available at the TCC before release of the trial treatment to a site. A separate Manual of Operating Procedures will detail the drug accountability system. The Investigator’s Brochure will detail labeling of the trial treatment and other processes for assuring adherence to Good Manufacturing Practice.

### Administration of trial treatment

Each treatment pack will contain:

· 4 × 500 mg ampoules of TXA or placebo

· 2 × sterile 10 mL syringe and 21FG needle

#### Loading dose

2 ampoules = 1 g – added to 100 mL sodium chloride 0.9% and infused over 10 minutes.

#### Maintenance dose

2 ampoules = 1 g – Added to 500 mL of any isotonic intravenous solution and infused at 120 mg/h (60 mL/h) for about 8 h.

The trial treatment injections should not be mixed with blood for transfusion, or infusion solutions containing penicillin or mannitol.

### Other treatments for traumatic brain injury

There is a wide spectrum of treatments for TBI. As the trial will be conducted worldwide, each participating site should follow its own clinical guidelines for the treatment of TBI patients. There is no need to withhold any clinically indicated treatment in this trial. TXA or placebo would be provided as an additional treatment to the usual management of TBI.

### Adverse events

TXA has a well documented safety profile. Although the Summary of Product Characteristics suggests that rare cases of thromboembolic events might be associated with TXA administration, there is no evidence that the TXA treatment regimen used in this trial is associated with an increased risk of vascular occlusive events. Nevertheless, data on vascular occlusive events and seizures will be collected as secondary outcomes and will be presented to the independent Data Monitoring Committee (DMC) for unblinded review.

### Definitions

**Adverse event:** Any untoward medical occurrence affecting a trial participant during the course of a clinical trial

**Serious Adverse Event (SAE):** A serious adverse event (experience) is any untoward medical occurrence that results in death, is life-threatening, requires inpatient hospitalization or prolongation of existing hospitalization, or results in persistent or significant disability/incapacity.

**Adverse Reaction:** An adverse event when there is at least a possibility that it is causally linked to a trial drug or intervention

**Serious Adverse Reaction (SAR):** SAE that is thought to be causally linked to a trial drug or intervention

**Suspected Unexpected Serious Adverse Reaction (SUSAR):** An unexpected occurrence of a SAR; there need only be an index of suspicion that the event is a previously unreported reaction to a trial drug or a previously reported but exaggerated or unexpectedly frequent adverse drug reaction.

Reporting of adverse events for this trial

Death, life-threatening complications, and prolonged hospital stay are pre-specified outcomes to be reported in this trial and also to the independent DMC. This clinical trial is being conducted in a critical emergency condition using a drug in common use. It is important to consider the natural history of the critical medical event affecting each patient enrolled, the expected complications of this event, and the relevance of the complications to TXA.

Adverse events to be reported using an adverse event reporting form will be limited to those not already listed as primary or secondary outcomes, but which might reasonably occur as a consequence of the trial drug. Events that are part of the natural history of the primary event of TBI or expected complications of TBI should not be reported as adverse events.

If an SAE, SAR or SUSAR occurs, a written report must be submitted within 24 h. Advice for investigators on reporting of adverse events is available by calling the TCC Emergency Helpline. The TCC will coordinate the reporting of all SAEs/SARs/SUSARs to all relevant Regulatory Agencies, Ethics Committees and local investigators as per local legal requirements.

### **Unblinding**

In general there should be no need to unblind the allocated treatment. If some contraindication to TXA develops after randomization (for example, clinical evidence of thrombosis), the trial treatment should simply be stopped and all usual standard care given. Unblinding should be done only in those rare cases when the clinician believes that clinical management depends importantly upon knowledge of whether the patient received TXA or placebo. In those few cases when urgent unblinding is considered necessary, a 24-h telephone service will be available and details provided in the Investigator’s Study File and wall posters. The caller will be told whether the patient received TXA or placebo. An unblinding report form should be completed by the investigator and sent to the Trial Coordinating Centre within 1 working day.

### Measures of outcome

After a patient has been randomized, outcome in hospital will be collected even if the trial treatment is interrupted or is not actually given. No extra tests are required but a short Outcome Form (Additional file 2: Form 2) will be completed 28 days after randomization, or at prior death or discharge from the randomizing hospital.

#### Primary outcome

The primary outcome is death in hospital within 28 days of injury (cause-specific mortality will also be recorded).

#### Secondary outcomes

a) Vascular occlusive events (myocardial infarction (MI), pulmonary embolism (PE), clinical evidence of deep vein thrombosis (DVT))

b) Stroke

c) Disability assessed using the DRS and Patient Orientated Outcome measures

d) Seizures

e) Neurosurgical intervention

f) Days in intensive care

g) Other adverse events will be described

#### Cost effectiveness analysis

A cost-utility analysis performed from a health care perspective will be conducted. Although the constraints of a large pragmatic trial reduce the scope for a comprehensive economic evaluation, the precise estimates of treatment effects from such studies are an important advantage. Data from the CRASH-3 trial will be used to populate a decision analytic model. The assessment of incremental cost effectiveness requires an estimate of health care costs and QALYs with and without administration of TXA. The incremental cost will be estimated using the data available at 28 days or discharge on ICU days, non-ICU days and health care interventions. If there are any significant differences in vascular events (PE, DVT, MI), stroke, or operative intervention, these can be used to refine the estimate of difference in cost. Life years gained will be modeled using the data on death or discharge in the first 28 days. Initially, it will be assumed that patients at discharge and those in hospital at 28 days have the life expectancy of their age-gender group. However, it will be important to explore alternative assumptions. Any significant differences in complications between the two treatment groups could be used to improve the estimate. Although CRASH-3 will not collect quality-of-life data directly, the detailed classification of the patient’s condition at discharge or 28 days can be used as the basis for a quality-of-life adjustment. Separate estimates of the incremental cost effectiveness ratio will be produced for the subgroups identified in the trial protocol. Some of the uncertainty surrounding the estimated cost effectiveness will be examined using deterministic and probabilistic sensitivity analysis.

### Data collection and management

This trial will be coordinated from the TCC at the London School of Hygiene & Tropical Medicine (LSHTM) and conducted in hospitals in low-, middle- and high-income countries. Data will be collected at each site by local investigators and sent to the TCC. Only data outlined on the entry, outcome, unblinding report and adverse event forms will be collected in this trial.

The entry form (Additional file 1: Form 1) will be used before randomization to confirm eligibility and collect baseline data. The outcome form (Additional file 2: Form 2) will be completed 28 days after randomization or at prior death or hospital discharge. These data will be collected from the patient’s routine medical records and no special tests will be required.

If a patient or their representative withdraws a previously given informed consent or refuses to consent for continuation in the trial, or if the patient dies and no consent is available, the patient’s data will be handled as follows:

· Data collected up to the point of withdrawal will be used in an intention-to-treat analysis.

· All data on adverse events, including those routinely collected as outcomes, will be collected and reported as required by the relevant authorities.

To allow for variation in available technology for data transfer, a variety of data collection methods will be used in the trial. Data will be collected by the investigator on paper case report forms (CRFs) and transmitted to the TCC either by fax or email or by entering the data directly into the trial database. Original paper CRFs will remain at each trial site. The data will be used in accordance with local law and ethics committee approval.

### Monitoring

ICH GCP section 5.18.3 states in regard to monitoring that *“the determination of the extent and nature of monitoring should be based on considerations such as the objective, purpose, design, complexity, blinding, size and endpoints of the trial. In general there is a need for on-site monitoring, before, during, and after the trial; however in exceptional circumstances the sponsor may determine that central monitoring in conjunction with procedures such as investigators training and meetings, and extensive written guidance can assure appropriate conduct of the trial in accordance with GCP. Statistically controlled sampling may be an acceptable method for selecting the data to be verified.”*

The CRASH-3 trial is a large, pragmatic, randomized, double-blind, placebo -controlled trial. The intervention (TXA) has marketing authorization in many countries and has been in clinical use for decades. Its safety profile is well established and no significant serious adverse events associated with its use have been identified. The trial will routinely collect data on adverse events which may theoretically be associated with this product and the condition under investigation, and these will be reviewed by the independent DMC. The trial procedures are based on routine clinical procedures and include (1) the intravenous administration of the trial drug using routine clinical use; (2) collecting routine clinical information from the medical records; and (3) informed consent. There are no complex procedures or interventions for the participants or investigators in this trial. Clinical management for underlying conditions will remain as per each hospital’s standard protocol. Based on these factors, the probability of harm or injury (physical, psychological, social or economic) occurring as a result of participation in this research study has been assessed as low in each of these categories. Based on the low risks associated with this trial, the Monitoring Procedure to assure appropriate conduct of the trial will utilize 100% central data monitoring in conjunction with procedures such as investigator training and meetings and written guidance. In addition, all data will be subject to statistical monitoring and approximately 10% of data will be subjected to on-site monitoring. Consent Forms will be monitored centrally by the TCC (where permission is given to do so). Investigators/institutions are required to provide direct access to source data/documents for trial-related monitoring, audits, ethics committee review and regulatory inspection. All trial-related and source documents must be kept for at least 5 years after the end of the trial.

### End of trial for participants

For the recruited patients the trial ends at death, hospital discharge or at 28 days follow-up, whichever occurs first. If during the treatment phase a patient develops an adverse event, the trial drug should be stopped, the patient treated in line with local procedures, and then followed up. The trial may be terminated early by the Trial Steering Committee (TSC).

The independent DMC may give advice/recommendation for the early termination of the trial but the TSC is responsible for the final decision.

### Analysis

The main analyses will compare all those allocated TXA versus those allocated placebo, on an ‘intention-to-treat’ basis, irrespective of whether they received the allocated treatment or not. Results will be presented as appropriate effect estimates (relative risks and absolute risks) with a measure of precision (95% CI). Subgroup analyses for the primary outcome will be based on time from injury to randomization, the severity of TBI (moderate or severe), the location of the intracranial bleeding, and baseline risk. Interaction tests will be used to test whether the effect of treatment (if any) differs across these subgroups. Unless there is strong evidence against the null hypothesis of homogeneity of effects (that is, *P* < 0.001), the overall relative risk will be considered as the most reliable guide to the approximate relative risks in all subgroups. Between-sites heterogeneity in effectiveness will also be explored.

A secondary analysis will be conducted in which the primary outcome will be adjusted by age, pupil reactivity, blood pressure and GCS. Because all secondary outcomes are non-fatal, the effect of TXA on these outcomes could be affected by competing risk by death. We will tackle this potential problem using the principal stratification method for studies with censoring due to death as proposed by Rubin [27]. A detailed Statistical Analysis Plan setting out full details of the proposed analyses will be finalized before the trial database is locked for final analysis.

### Sponsorship and trial management

The CRASH-3 trial is sponsored by the LSHTM and its responsibilities coordinated by the TCC. The TCC may delegate responsibilities to third parties which will be outlined in relevant agreements. The responsibilities of the TCC will be overseen by the Trial Management Group (TMG).

### Indemnity

LSHTM accepts responsibility attached to its sponsorship of the trial and, as such, would be responsible for claims for any non-negligent harm suffered by anyone as a result of participating in this trial. The indemnity is renewed on an annual basis and LSHTM assures that it will continue renewal of the indemnity for the duration of this trial.

### Protocol development

The Protocol Committee consists of the following investigators who are responsible for the development of, and agreeing to, the final protocol. Subsequent changes to the final protocol will require the agreement of the TSC.

· Chief investigator: Professor Ian Roberts

· Clinical experts: Professor Yashbir Dewan, Dr Jorge H Mejía-Mantilla, Dr Edward Komolafe, Dr Pablo Perel

· Trial management: Ms Haleema Shakur

· Statistician: Dr Phil Edwards

### Independent Data Monitoring Committee

· Dr Samuel C. Ohaegbulam, Neurosurgeon-in-Chief, Memfys Hospital for Neurosurgery, Nigeria, Neurosurgery

· Professor Anthony Rodgers, George Institute, Australia, Clinical Trials

· Professor Mike Clarke, University of Belfast, UK, Epidemiology and statistics

To provide protection for study participants, an independent DMC has been appointed for this trial to oversee the safety monitoring. The DMC will review, on a regular basis, accumulating data from the ongoing trial and advise the TSC regarding the continuing safety of current participants and those yet to be recruited, as well as reviewing the validity and scientific merit of the trial.

The DMC composition, name, title and address of the chairman and of each member, will be given in the DMC Charter. Membership includes expertise in the relevant field of study, statistics and research study design. An independent statistician will be appointed to provide the analysis service required by the DMC. The DMC Charter includes, but is not limited to, defining:

a) the schedule and format of the DMC meetings

b) the format for presentation of data

c) the method and timing of providing interim reports

d) stopping rules

#### Standard operating procedures

The DMC has the responsibility for deciding whether, while randomization is in progress, the unblinded results (or the unblinded results for a particular subgroup), should be revealed to the TSC. The DMC Charter states that they will do this if, and only if, the following two conditions are satisfied: (1) the results provide proof beyond reasonable doubt that treatment is on balance either definitely harmful or definitely favourable for all, or for a particular category of participants in terms of the major outcome; and (2) the results, if revealed, would be expected to substantially change the prescribing patterns of clinicians who are already familiar with any other trial results that exist. Exact criteria for “proof beyond reasonable doubt” are not, and cannot be, specified by a purely mathematical stopping rule, but they are strongly influenced by such rules. The DMC Charter is in agreement with the Peto-Haybittle stopping rule whereby an interim analysis of a major endpoint would generally need to involve a difference between treatment and control of at least three standard errors to justify premature disclosure [28,29]. An interim subgroup analysis would, of course, have to be even more extreme to justify disclosure. This rule has the advantage that the exact number and timing of interim analyses need not be pre-specified. In summary, the stopping rules require extreme differences to justify premature disclosure, and involve an appropriate combination of mathematical stopping rules and scientific judgment.

### Trial steering committee

· Professor Peter Sandercock (Chair), Western General Hospital; Professor of Medical Neurology, Director, Edinburgh Neuroscience, University of Edinburgh, UK (randomized control trials, conduct of large scale international trials)

· HB Hartzenberg, Tygerberg Academic Hospital, Faculty of Health Sciences, University of Stellenbosch, South Africa; Professor and Head of Neurosurgery (previous President of the Society of Neurosurgeons of South Africa)

· Amy Aeron-Thomas, Executive Director, RoadPeace, the national charity for road crash victims, London, UK (Expertise includes developing national road safety action plans, costing crashes and documenting their socio-economic impact on families. Road safety pilot project in Nigeria, intended to improve compensation for road crash victims and increase awareness of the road traffic injury burden.)

· Manjul Joshipura, Scientist (trauma care), World Health Organization, Geneva, Switzerland (Scientific leadership and technical support in the field of trauma care to the WHO; previous Director of Academy of Traumatology, India)

· Ian Roberts, London School of Hygiene & Tropical Medicine, London, UK; Professor of Epidemiology (randomized control trials; conduct of large scale international trials)

· Pablo Perel, London School of Hygiene & Tropical Medicine, London, UK; Clinical Lecturer (randomized control trials; trial methodology)

· Haleema Shakur, London School of Hygiene & Tropical Medicine, London, UK; Senior Lecturer (trial methodology; randomized control trials; conduct of large scale international trials)

The role of the TSC is to provide overall supervision of the trial. In particular, the TSC will concentrate on the progress of the trial, adherence to the protocol, patient safety and consideration of new information. The TSC must be in agreement with the final protocol and, throughout the trial, will take responsibility for:

a) major decisions such as a need to change the protocol for any reason

b) monitoring and supervising the progress of the trial

c) reviewing relevant information from other sources

d) considering recommendations from the DMC

e) informing and advising the TMG on all aspects of the trial

The Steering Committee consists of people with experience in clinical trials, traumatic brain injury research and patient representatives. Face-to-face meetings will be held at regular intervals determined by need, but no less than once a year. A TSC Charter will be agreed at the first meeting and will detail how the committee will conduct its business.

When outcome data are available for 500 trial participants, the TSC will review the rate of recruitment into the trial and the overall event rates. The TSC will consider the extent to which the rate of recruitment and the event rates correspond to those anticipated before the trial and will take whatever action is needed in light of this information.

### Advisory committees

An *ad hoc* advisory group was established at the protocol development stage of the CRASH-3 trial with the responsibility of ensuring the protocol was appropriate to populations in a wide variety of settings. Clinicians and clinical trialists (including neurosurgeons and other trauma specialists) from UK, Colombia, India and Nigeria were consulted during face-to-face meetings in each country and their input was incorporated in the final protocol. The members of the advisory group are listed at the trial website (http://crash3.lshtm.ac.uk/ª ).

In addition, an International Advisory Committee (IAC) will be convened to fulfil two roles:

(a) to advise the TMG on matters relevant to the trial, and

(b) to enable appropriate representation of each country’s views on the trial.

The role of the IAC is advisory only. The IAC will constitute the National Coordinators from participating countries and other individuals with relevant expertise. The IAC will be chaired by the Chair of the TMG. New members will be added as new countries join the trial and National Coordinators are appointed. The IAC will provide advice and comments to the TMG. The TMG will inform the TSC accordingly on matters raised by the IAC that relate either to the protocol or which might have an impact on the progress of the trial. The TMG will convey any relevant comments from the IAC to the TCC on matters relating to the day-to-day management of the trial. An important function of the IAC is to facilitate the sharing of experience and best practice between its members on how best to conduct the trial efficiently within each country and how to overcome barriers to progress. The IAC’s chief role is therefore to report on the progress of the trial within each country and to provide advice to the TMG, TSC and TCC in order to maximize the efficiency of the trial’s conduct, and hence the chances of completing the trial on time and within budget.

### Collaborators’ responsibilities

Coordination within each participating hospital will be through a local Principal Investigator whose responsibility will be detailed in an agreement in advance of starting the trial and will include:

· Ensure all necessary approvals are in place prior to starting the trial

· Delegate trial-related responsibilities only to suitably trained and qualified personnel

· Train relevant medical and nursing staff who see TBI patients and ensure that they remain aware of the state of the current knowledge, the trial and its procedures (there are wall charts, pocket summaries and training presentations to assist with this)

· Agree to comply with the final trial protocol and any relevant amendments

· Ensure that all patients with TBI are considered promptly for the trial

· Ensure consent is obtained in line with local approved procedures

· Ensure that the patient entry and outcome data are completed and transmitted to the TCC in a timely manner

· Ensure the Investigator’s Study File is up to date and complete

· Ensure all adverse events are reported promptly to the TCC

· Be accountable for trial treatments at their site

· Ensure the trial is conducted in accordance with ICH GCP and fulfils all national and local regulatory requirements

· Allow access to source data for monitoring, audit and inspection

· Be responsible for archiving all original trial documents, including the data forms, for 5 years after the end of the trial

### Trial management group and Trial Coordinating Centre responsibilities

The TMG will consist of at least the following members: Chief Investigator, a trial manager and a clinical expert. The TCC will act on behalf of the Sponsor and will be responsible to the TMG to ensure that all Sponsor’s responsibilities are carried out. The responsibilities will include (but are not limited to):

· Reporting to the TSC

· The day-to-day management of the trial

· Ensuring that all relevant procedures for the conduct of the trial are in place

· Advising the TCC staff on specific aspects as required

· Maintaining the Trial Master File

· Identifying trial sites

· Confirming all approvals are in place before release of trial treatment and the start of the trial at a site

· Providing training about the trial

· Providing study materials

· Acting as the data management centre

· Providing a 24-h advice and unblinding service

· Giving collaborators regular information about the progress of the study

· Responding to questions (for example, from collaborators) about the trial

· Ensuring data security and quality and observe data protection laws

· Safety reporting

· Ensuring the trial is conducted in accordance with the ICH GCP

· Statistical analysis

· Publication of trial results

### Contacting the Trial Coordinating Centre in an emergency

For urgent enquiries, adverse event reporting and unblinding queries, investigators can contact the 24-h telephone service provided by the TCC. A central telephone number is given in the Investigator’s Study File and posters.

### Publication and dissemination of results

All efforts will be made to ensure that the trial protocol and results arising from the CRASH-3 trial are published in an established peer-reviewed journal. At least one publication of the main trial results will be made. On reflection this information is not needed – too much detail. Links to the publication will be provided in all applicable trial registers. Dissemination of results to patients will take place via the media, trial website (http://crash3.Lshtm.ac.uk^a^) and relevant patient organizations. In addition, participants and their families will be made aware of the trial results if requested. Collaborating investigators will play a vital role in disseminating the results to colleagues and patients. The success of the trial will be dependent entirely upon the collaboration of the nurses and doctors in the participating hospitals and those who hold key responsibility for the trial. Hence, the credit for the study will be assigned to the key collaborator(s) from each participating site, as it is crucial that those taking credit for the work have actually carried it out. The results of the trial will be reported first to trial collaborators. As a large number of hospitals in many countries will contribute to this trial, individual countries or sites cannot restrict the publication of the manuscript relating to the outcomes of this trial. Anonymous data for this trial will be made available for free use at http://freebird.lshtm.ac.uk.^b^

### Financial support

The JP Moulton Charitable Trust, UK, is funding the run-in costs for this trial and up to 500 patients’ recruitment. Full funding is being sought from public funding organizations for the main trial. Funding for this trial covers meetings and central organizational costs only. The design and management of the study are entirely independent of the manufacturers of TXA or the funders.

Large trials of drugs such as TXA, involving many hospitals, are important for future patients, but are practicable only if those collaborating in them do so without payment (except for recompense of any minor local costs that may arise). Agreement for repayment of local costs will be made in advance. This trial will not generate any intellectual property for the Sponsor or collaborating institutions. The trial plans to include over 250 hospitals in about 40 countries. Review by each Ethics Committee and Regulatory Agency would create a substantial financial burden which could limit the conduct of the trial. We request that payment for review of the protocol by each Committee be waived or set at a reasonable rate to reflect the actual cost of reviewing the trial protocol.

## Trial status

Ethics approval obtained from several institutions. National ethics and regulatory approvals are in progress in 10 countries. Patient recruitment is planned to take place in over 30 countries and is due to start in April 2012. End of recruitment is planned for 31 December 2016 with end of follow-up due 31 January 2017. Further information is available at http://crash3.lshtm.ac.uk/

## Abbreviations

CI, Confidence interval; CRF, Case report form; CT, Computerized tomography; DMC, Data Monitoring Committee; DRS, Disability Rating Scale; DVT, Deep vein thrombosis; GCP, Good Clinical Practice; GCS, Glasgow Coma Score; GMP, Good Manufacturing Practice; HPLC, High-performance liquid chromatography; IAC, International Advisory Committee; ICMJE, International committee for medical journal editors; LSHTM, London School of Hygiene & Tropical Medicine; MI, Myocardial infarction; PE, Pulmonary embolism; PSF, Product Specification File; QALY, Quality Adjusted Life Year; QP, Qualified person; RR, Relative Risk; SAE, Serious adverse event; SAR, Serious adverse reaction; STATA, ; SUSAR, Suspected unexpected serious adverse reaction; TCC, Trial Coordinating Centre; TBI, Traumatic brain injury; TMG, Trial Management Group; tPA, Tissue plasminogen activator; TSC, Trial Steering Committee; TXA, Tranexamic acid; WHO, World Health Organization.

## Competing interests

The authors declare that they have no competing interests.

## Authors’ contributions

IR, HS, PP, YD, EK and JM have made substantial contributions to the concept and design of the trial. They were all involved in drafting the protocol, revising it critically for important intellectual content and have given approval of the final version to be used in this trial.

## Supplementary Material

Additional file 1 Form 1. Entry form. Click here for file

Additional file 2 Form 2. Ou form. Click here for file

Additional file 3 Form 3. Brief intcomeformation leaflet for relatives. Click here for file

Additional file 4 Form 4. Information sheet for the patient and representative. Click here for file

Additional file 5 Form 5. Consent form for the patient and representative. Click here for file
