# Supplementary material for: CRASH-3 - tranexamic acid for the treatment of significant traumatic brain injury: study protocol for an international randomized, double-blind, placebo-controlled trial
Source: Trials. 2012 Jun 21;13:87. doi: 10.1186/1745-6215-13-87 (PMC3481366; doi:10.1186/1745-6215-13-87)
Supplement: Additional file 1 — Form 1. Entry form. [file 1745-6215-13-87-S1.pdf]

ENTRY FORM

PLEASE COMPLETE 1-16 BEFORE RANDOMISING THE PATIENT

ABOUT YOUR HOSPITAL *(please ensure all information below is contained in the medical records)*

**1.** Country	
**2.** Hospital code *(in your Study File)*	

ABOUT THE PATIENT

**3.** Patient's initials *(first name/last name)*			**4.** Patient hospital ID		
**5.** Age *(years – approximate if unknown)*			**6.** Sex *(circle)*	**MALE**	**FEMALE**

ABOUT THE INJURY AND PATIENT'S CONDITION

**7.** Time since injury *(insert hours)*		*Best estimate from history*	
**8.** Systolic Blood Pressure		*mmHg (most recent measurement prior to randomisation)*	

9. Glasgow Coma Score (GCS) *(circle one response for each category)*

First measurement in hospital of GCS (if unknown give value at randomisation)

9A–EYE OPENING	9B–MOTOR RESPONSE	9C–VERBAL RESPONSE
4 Spontaneous	6 Obeys commands	5 Orientated
3 To sound	5 Localising	4 Confused speech
2 To pain	4 Normal flexion	3 Words
1 None	3 Abnormal flexion	2 Sounds
	2 Extending	1 None
	1 None	

*IF GCS MORE THAN 12 AND NO CT SCAN AVAILABLE – **DO NOT RANDOMISE***

*IF GCS MORE THAN 12, CT SCAN **IS** AVAILABLE AND INTRACRANIAL BLEEDING=YES – **RANDOMISE***

**10.** This GCS is *(circle one)*	**BEFORE**	**AFTER**	intubation/sedation		
**11.** Pupil reaction	**BOTH REACT**		**ONE REACTS**	**NONE REACT**	**UNABLE TO ASSESS**
**12.** Any significant extracranial bleeding?	**YES**	**NO**	*Patients with extracranial trauma who are likely to need an early blood transfusion in the view of the attending doctor after taking into account mechanism of injury, findings from secondary survey, physiology and response to fluid infusion – **DO NOT RANDOMISE***		
**13.** Any intracranial bleeding on CT scan (before randomisation)? *(circle one)*	**YES**	**NO**	**NO CT SCAN AVAILABLE**	*IF CT SCAN AVAILABLE AND INTRACRANIAL BLEEDING=NO – **DO NOT RANDOMISE***	

14. Location of intracranial haemorrhage on CT Scan *(circle one response for each line)*

a) Epidural	**YES**	**NO**
b) Subdural	**YES**	**NO**
c) Subarachnoid	**YES**	**NO**
d) Parenchymal	**YES**	**NO**
e) Intraventricular	**YES**	**NO**

RANDOMISATION INFORMATION

Eligible if adult, with TBI, no significant extracranial bleeding, within 8h of injury (GCS=12 or less, or any intracranial haemorrhage on CT scan)

**15.** Eligible? *(circle)*	**YES**	*Get the lowest available number treatment pack and follow instructions*	**NO**	*Do not randomise, record on screening log*
**16.** Consent process for entry used? *(circle)*	**WAIVER**	**OTHER REPRESENTATIVE**	**RELATIVE**	

**17.** Insert treatment pack number here	**BOX**					**PACK**	
**18.** Date of randomisation	*day*	*month*	*year*	**19.** Time of randomisation *(24-hour clock)*	*hours*	*minutes*	
**20.** Name of person randomising				**21.** Signature			

SEE GUIDANCE OVERLEAF

DATA FORMS GUIDANCE

AFTER COMPLETING THIS PAPER FORM PLEASE SEND THE DATA BY ANY METHOD LISTED:

- Enter these data directly into the trial database (username and password required)
- Upload as a secure scanned document (see Study File for details)
- Fax to +44 20 7299 4663

PLEASE STORE THE ORIGINAL FORM IN THE INVESTIGATOR'S STUDY FILE

PLEASE GIVE A COPY OF THIS COMPLETED FORM TO THE PERSON RESPONSIBLE FOR COMPLETING THE OUTCOME FORM AT YOUR HOSPITAL.

FOR UNBLINDING, ADVICE ON SERIOUS ADVERSE EVENT REPORTING AND OTHER URGENT ENQUIRIES PLEASE TELEPHONE +44(0)7768 707500
