# Supplementary material for: CRASH-3 - tranexamic acid for the treatment of significant traumatic brain injury: study protocol for an international randomized, double-blind, placebo-controlled trial
Source: Trials. 2012 Jun 21;13:87. doi: 10.1186/1745-6215-13-87 (PMC3481366; doi:10.1186/1745-6215-13-87)
Supplement: Additional file 2 — Form 2. Ou form. [file 1745-6215-13-87-S2.pdf]

CRASH
Clinical Randomisation of an Antifibrinolytic in Significant Head Injury

OUTCOME FORM

COMPLETE AT DISCHARGE FROM THE RANDOMISING HOSPITAL, DEATH IN HOSPITAL OR 28 DAYS AFTER INJURY, WHICHEVER OCCURS FIRST

Attach here a sticker from the lid of the treatment pack or write box/pack number below:

[ ][ ][ ][ ] / [ ][ ]

1. HOSPITAL

(Hospital code)

2. PATIENT

a) BOX [ ][ ][ ][ ] b) PACK [ ][ ] c) INITIALS [ ][ ]

3. OUTCOME

3.1 DEATH IN HOSPITAL

a) Date of death

DAY (DD) | MONTH (MM) | YEAR (YYYY)

b) Time of death

HOUR (HH) | MIN (MM)

c) Primary Cause of death *(tick one option)*

- ☐ Head injury
- ☐ Bleeding
- ☐ Pulmonary embolism
- ☐ Stroke
- ☐ Myocardial Infarction
- ☐ Multi organ failure
- ☐ Other/describe here (only one)____________________________________

3.2 PATIENT ALIVE

a) Still in this hospital now *(28 days after randomisation)* – Date

DAY (DD) | MONTH (MM) | YEAR (YYYY)

b) Discharged to another hospital – Date of discharge

DAY (DD) | MONTH (MM) | YEAR (YYYY)

c) Discharged home – Date of discharge

DAY (DD) | MONTH (MM) | YEAR (YYYY)

3.3 IF ALIVE – DISABILITY RATING SCALE *(tick one response for each box) – see overleaf for guidance*

a) EYE OPENING
- ☐ Spontaneous
- ☐ To Speech
- ☐ To Pain
- ☐ None

b) COMMUNICATION ABILITY
- ☐ Oriented
- ☐ Confused
- ☐ Inappropriate
- ☐ Incomprehensible
- ☐ None

c) MOTOR RESPONSE
- ☐ Obeying
- ☐ Localizing
- ☐ Withdrawing
- ☐ Flexing
- ☐ Extending
- ☐ None

d) FEEDING *(cognitive ability only)*
- ☐ Complete
- ☐ Partial
- ☐ Minimal
- ☐ None

e) TOILETING *(cognitive ability only)*
- ☐ Complete
- ☐ Partial
- ☐ Minimal
- ☐ None

f) GROOMING *(cognitive ability only)*
- ☐ Complete
- ☐ Partial
- ☐ Minimal
- ☐ None

g) LEVEL OF FUNCTIONING *(physical, mental, emotional or social function)*
- ☐ Completely independent
- ☐ Independent in special environment
- ☐ Mildly dependent – limited assistance
- ☐ Moderately dependent – moderate assistance
- ☐ Markedly dependent – assist all major activities, all times
- ☐ Totally dependent – 24-hour nursing care

h.'EMPLOYABILITY' *(as a full time worker, homemaker, or student)*
- ☐ Not restricted
- ☐ Selected jobs, competitive
- ☐ Sheltered workshop, non-competitive
- ☐ Not employable

3.4 IF ALIVE: *Assessed by doctor/nurse/relative based on their knowledge of the patient, or patient if able (tick one response for each box)*

SEE GUIDANCE OVERLEAF

a) WALKING
- ☐ No problems
- ☐ Some problems
- ☐ Confined to bed

b) WASHING / DRESSING
- ☐ No problems
- ☐ Some problems
- ☐ Unable

c) PAIN / DISCOMFORT
- ☐ None
- ☐ Moderate
- ☐ Extreme

d) ANXIETY / DEPRESSION
- ☐ None
- ☐ Moderate
- ☐ Extreme

e) AGITATION / AGGRESSION
- ☐ None
- ☐ Moderate
- ☐ Extreme

f) FATIGUE
- ☐ None
- ☐ Moderate
- ☐ Extreme

4. MANAGEMENT

a) DAYS IN INTENSIVE CARE UNIT *(if no ICU or not admitted to ICU, write '0' here)*		
**b) TYPE OF NEUROSURGICAL OPERATION**		
i) Haematoma evacuation	YES	NO
ii) Other	YES	NO
**c) BLOOD LOSS DURING NEUROSURGICAL OPERATION**		
Estimated Volume (ml)		

6. COMPLICATIONS

(circle one option on every line)

Pulmonary embolism	YES	NO
Deep vein thrombosis	YES	NO
Stroke	YES	NO
Myocardial infarction	YES	NO
Renal failure	YES	NO
Sepsis	YES	NO
Seizure	YES	NO
Gastro intestinal bleeding	YES	NO

5. TRIAL TREATMENT

a) Loading dose given	YES	NO
b) Maintenance dose given	YES	NO

7. OTHER COMPLICATIONS YES NO

IF YES, REPORT AS PER PROTOCOL USING ADVERSE EVENT FORM

8. PERSON COMPLETING FORM

THE PRINCIPAL INVESTIGATOR IS RESPONSIBLE FOR ALL DATA SUBMITTED

a) Name		b) Position	
c) Signature		d) Date	

GUIDANCE – HOW TO COMPLETE THE DISABILITY RATING SCALE, QUESTION 3.3 OVERLEAF

A. EYE OPENING

0–SPONTANEOUS: eyes open with sleep/wake rhythms indicating active arousal mechanisms, does not assume awareness

1–TO SPEECH AND/OR SENSORY STIMULATION: response to any verbal approach, spoken/shouted, not necessarily the command to open the eyes. Also response to touch, mild pressure

2–TO PAIN: tested by a painful stimulus

3–NONE: no eye opening even to painful stimulation

B. COMMUNICATION ABILITY

0–ORIENTED: implies awareness of self and the environment. Patient able to tell you a) who he is; b) where s/he is; c) why he is there; d) year; e) season; f) month; g) day; h) time of day

1–CONFUSED: attention can be held and patient responds to questions but responses are delayed and/or indicate varying degrees of disorientation and confusion

2–INAPPROPRIATE: intelligible articulation but speech is used only in an exclamatory or random way (such as shouting and swearing); no sustained communication exchange is possible

3–INCOMPREHENSIBLE: moaning, groaning or sounds without recognizable words, no consistent communication signs

4–NONE: no sounds or communications signs from patient

C. MOTOR RESPONSE

0–OBEYING: obeying command to move finger on best side. If no response or not suitable try another command such as “move lips,” “blink eyes,” etc. Do not include grasp or other reflex responses

1–LOCALIZING: a painful stimulus at more than one site causes limb to move (even slightly) in an attempt to remove it. It is a deliberate motor act to move away from or remove the source of noxious stimulation. If there is doubt as to whether withdrawal or localization has occurred after 3 or 4 painful stimulations, rate as localization

2–WITHDRAWING: any generalized movement away from a noxious stimulus that is more than a simple reflex response

3–FLEXING: painful stimulation results in either flexion at the elbow, rapid withdrawal with abduction of the shoulder or a slow withdrawal with adduction of the shoulder. If there is confusion between flexing and withdrawing, then use pinprick on hands

4–EXTENDING: painful stimulation results in extension of the limb

5–NONE: no response can be elicited. Usually associated with hypotonia. Exclude spinal transection as an explanation of lack of response; be satisfied that an adequate stimulus has been applied

D.FEEDING, E.TOILETING, F.GROOMING (COGNITIVE ABILITY ONLY FOR EACH)

Does the patient show awareness of how and when to perform this activity? Ignore motor disabilities that interfere with carrying out this function (rated under Level of Functioning below.)

- Rate best response for toileting based on bowel and bladder behavior
- Grooming refers to bathing, washing, brushing of teeth, shaving, combing or brushing of hair and dressing

0–COMPLETE: continuously shows awareness that he knows how to feed and can convey unambiguous information that he knows when this activity should occur

1–PARTIAL: intermittently shows awareness that he knows how to carry out this activity and/or can intermittently convey reasonably clearly information that he knows when the activity should occur

2–MINIMAL: shows questionable or infrequent awareness that he knows in a primitive way how to carry out this activity and/or shows infrequently by certain signs, sounds, or activities that he is vaguely aware when the activity should occur

3–NONE: shows virtually no awareness at any time that he knows how to carry out this activity and cannot convey information by signs, sounds, or activity that he knows when the activity should occur

G.LEVEL OF FUNCTIONING (PHYSICAL, MENTAL, EMOTIONAL OR SOCIAL FUNCTION)

0–COMPLETELY INDEPENDENT: able to live as he wishes, requiring no restriction due to physical, mental, emotional or social problems

1–INDEPENDENT IN SPECIAL ENVIRONMENT: capable of functioning independently when needed requirements are met (mechanical aids)

2–MILDLY DEPENDENT: able to care for most of own needs but requires limited assistance due to physical, cognitive and/or emotional problems (e.g., needs non-resident helper)

3–MODERATELY DEPENDENT: able to care for self partially but needs another person at all times (person in home)

4–MARKEDLY DEPENDENT: needs help with all major activities and the assistance of another person at all times

5–TOTALLY DEPENDENT: not able to assist in own care and requires 24-hour nursing care

H.’EMPLOYABILITY’(AS A FULL TIME WORKER, HOMEMAKER, OR STUDENT)

0–NOT RESTRICTED: can compete in the open market for a relatively wide range of jobs commensurate with existing skills; or can initiate, plan execute and assume responsibilities associated with homemaking; or can understand and carry out most age relevant school assignments

1–SELECTED JOBS, COMPETITIVE: can compete in a limited job market for a relatively narrow range of jobs because of limitations of the type described above and/or because of some physical limitations; or can initiate, plan, execute and assume many but not all responsibilities associated with homemaking; or can understand and carry out many but not all school assignments

2–SHELTERED WORKSHOP, NON-COMPETITIVE: cannot compete successfully in a job market because of limitations described above and/or because of moderate or severe physical limitations; or cannot without major assistance initiate, plan, execute and assume responsibilities for homemaking; or cannot understand and carry out even relatively simple school assignments without assistance

3–NOT EMPLOYABLE: completely unemployable because of extreme psychosocial limitations of the type described above, or completely unable to initiate, plan, execute and assume any responsibilities associated with homemaking; or cannot understand or carry out any school assignments

GUIDANCE – HOW TO COMPLETE THE ASSESSMENT IN QUESTION 3.4 OVERLEAF

To indicate which statement best describes the patient’s status on discharge or day 28 (if still in hospital), place a tick ✓ in one box in each group. **Do not tick more than one box in each group.**
