# Supplementary material for: CRASH-3 - tranexamic acid for the treatment of significant traumatic brain injury: study protocol for an international randomized, double-blind, placebo-controlled trial
Source: Trials. 2012 Jun 21;13:87. doi: 10.1186/1745-6215-13-87 (PMC3481366; doi:10.1186/1745-6215-13-87)
Supplement: Additional file 3 — Form 3. Brief intcomeformation leaflet for relatives. [file 1745-6215-13-87-S3.pdf]

BRIEF INFORMATION FOR THE PATIENT'S FAMILY

THE CRASH-3 TRIAL

Tranexamic acid for the treatment of significant traumatic brain injury; an international randomised, double blind placebo controlled trial

Your relative has a head injury that needs urgent care. He/she will get all the usual emergency care for head injury that we provide at this hospital. As well as this, we would like to include him/her in an international study. This study will see whether a drug called tranexamic acid reduces bleeding inside the head after head injury. We hope that the drug will lead to a better recovery. We know that the drug reduces bleeding in other types of severe injury and without side effects, but as yet we don't know if it works in head injury.

As part of the study, your relative will receive an injection into a vein followed by a drip over eight hours. Half the patients in the study will get the tranexamic acid and half a dummy drug (a liquid which does not contain tranexamic acid). We will not know until the end of the study who received which treatment. We will need to collect some information about your relative's medical condition and send it to a central office in London.

If you would like to know more about our study now, then we will tell you. But otherwise we will tell you more about it later. Are you happy for us to go ahead with the study treatment?

Yes, I am happy for you to go ahead.

Name: ____

Signature: ____

Relationship to Patient: ____

Date: ____

(Complete only where required)
