# Supplementary material for: CRASH-3 - tranexamic acid for the treatment of significant traumatic brain injury: study protocol for an international randomized, double-blind, placebo-controlled trial
Source: Trials. 2012 Jun 21;13:87. doi: 10.1186/1745-6215-13-87 (PMC3481366; doi:10.1186/1745-6215-13-87)
Supplement: Additional file 4 — Form 4. Information sheet for the patient and representative. [file 1745-6215-13-87-S4.pdf]

[HOSPITAL CONTACT DETAILS]

INFORMATION FOR REPRESENTATIVES AND PATIENTS
THE CRASH-3 TRIAL

**Trial title**	Tranexamic acid for the treatment of significant traumatic brain injury; an international randomised, double blind placebo controlled trial
**Trial site number**	
**Leaflet version**	

This hospital is taking part in an international study to find better treatments for head injury.
(One of the following two options apply to you)

(1) As a patient representative: This leaflet gives information about the study to help you to make a decision on the patient's behalf.

(2) As the patient: After your head injury, you got all the usual emergency care for head injury that we provide at this hospital. The decision was also taken for you to be part of an international study to find better treatments for head injury. Because of your injury you could not make this decision yourself but now that your condition has improved, we would like to tell you about the study and ask whether you want to stay part of it.

Before you decide, it is important that you know why the study is being done and what it involves. Please read the information below and ask as many questions as you like before deciding. This leaflet explains why we are doing the study and outlines the benefits and risks of taking part. The Doctor or Nurse will be happy to talk to you about the study and answer any questions.

1) What is the purpose of this study?

In this hospital, patients with head injury are given the usual emergency treatments. The aim of this study is to find a better treatment to improve recovery. We hope that the study treatment (tranexamic acid) will prevent or reduce bleeding into the head after head injury and so lead to better outcomes. In general, there are no medical reasons why tranexamic acid should not be given to patients with head injury but if the doctor felt that it was not suitable in your particular case they would not include you (the patient) in the study. We hope that the treatment will do more good than harm but we don't yet know this.

2) Why have you/the patient been chosen to take part?

This study is being done to see if a drug called tranexamic acid improves outcome after head injury. You (the patient) have been included because you suffered a serious head injury which could cause bleeding into the brain. You (the patient) are one of about 10,000 patients with head injury from all around the world taking part in this study.

3) A patient cannot be in this study if:

- the doctor thinks there is a particular reason why tranexamic acid definitely **should not** be given
- the doctor thinks there is a particular reason why tranexamic acid definitely **should** be given
- he/she is not an adult
- he/she was injured more than 8 hours before arriving at the hospital

4) What does taking part in this study involve?

All the usual emergency treatments for head injury are given. We do not know whether giving tranexamic acid on top of all the other treatments will help or not, so half the patients in the study will get tranexamic acid and the other half will get a placebo (dummy treatment). The treatment will be/was given over a period of eight hours. The choice of what to give (active treatment or dummy treatment) is made randomly (like a lottery), and you/the patient will have an equal chance of receiving either one. The doctors looking after you/the patient do not know whether you/the patient received tranexamic acid or the dummy medicine. You/the patient will not need to undergo any extra tests or spend any extra time in hospital as a result of taking part in this study. The study treatment is free.

5) What are the possible risks of being in the study?

Tranexamic acid is not a new drug. It has been used for years to reduce bleeding after operations and more recently to treat other types of serious injury. It works by stopping the breakdown of the blood clots which are needed to control bleeding. Studies have shown that it does not cause unwanted clotting and there are no serious side effects with short term use. However, the doctor will monitor you/the patient closely and will report to the study organisers if there are any unexpected problems.

6) What are the possible benefits of being in the study?

We hope that tranexamic acid will help reduce bleeding into the head after head injury, which is a common cause of death and disability after head injury. The knowledge that we gain from this study will help people with similar injuries in the future.

7) What if I don't want to be a part of this study anymore?

You can always withdraw from the study at any time. You just need to say for example *"I've decided I don't want to be in this study now"*. We hope that you will let us use information about how you/the patient got on, but if you do not want us to use it please tell the doctor.

8) Will the information you collect be kept private?

All information about you/the patient and the injury will be kept private. The only people allowed to look at the information will be the doctors running the study and the staff at the Trial Coordinating Centre at LSHTM (University of London) and the regulatory authorities who check that the study is being carried out correctly. Your doctor will send brief details about you/the patient to the Trial Coordinating Centre at LSHTM. Personal information will be used in strict confidence by the people working on the study and will not be released under any circumstance. We will publish the results of the study in a medical journal so that other doctors can benefit from the knowledge, but your/the patient's personal information will not be included and there will be no way that you/the patient can be identified. Data with no personal information attached will be made available for use by other researchers and the public.

The Trial Coordinating Centre may want to collect or copy some trial documents which will have your name and will include the signed Consent Form. This will help them to ensure that the trial is being carried out correctly.

9) Who can you/the patient contact about any questions or problems?

If you have any questions or concerns about any aspect of this study, you should ask to speak with the study doctors who will do their best to answer your questions. Dr [insert name] is in charge of this study at this hospital. You can contact the doctor at:

Address	
Telephone	

If you are still unhappy and wish to complain formally, you can do this through the hospital's complaints procedure. Please ask the researchers for details.

This study is co-ordinated by doctors and a trial team at the London School of Hygiene & Tropical Medicine (LSHTM) at the University of London.

10) Who has reviewed the study?

To protect your interests, all studies conducted at this hospital are looked at by an independent group of people called a Research Ethics Committee. This study has been reviewed and has been given a favourable ethical opinion by [insert name] Research Ethics Committee.

11) What happens afterwards?

We would like to hear if you/the patient develop(s) any medical problems after discharge from this hospital and at any time up to 28 days. You will be given a card with the contact details of the research doctor at this hospital, which should be kept in a safe place and presented to anyone who may be treating you/the patient for any illness.

If you would like to have a copy of the final results of this study, please let the research doctor know and s/he will ensure you receive a copy when the results are published. You may also visit the trial website to keep up to date with the progress of the trial: **http://crash3.Lshtm.ac.uk**

12) What else do you need to know?

- The study is organised by the University of London and funded by public and charitable funds, not the makers of tranexamic acid.
- LSHTM (University of London) as the organisers of the study accepts responsibility attached to its sponsorship of the study and, as such, would be responsible for claims for any non-negligent harm suffered by anyone as a result of participating in this study.
- We will ask you to sign a separate consent form and give you a copy to keep and you can also keep this information sheet.
