# Supplementary material for: CRASH-3 - tranexamic acid for the treatment of significant traumatic brain injury: study protocol for an international randomized, double-blind, placebo-controlled trial
Source: Trials. 2012 Jun 21;13:87. doi: 10.1186/1745-6215-13-87 (PMC3481366; doi:10.1186/1745-6215-13-87)
Supplement: Additional file 5 — Form 5. Consent form for the patient and representative. [file 1745-6215-13-87-S5.pdf]

[HOSPITAL CONTACT DETAILS]

CONSENT FORM FOR PATIENT AND REPRESENTATIVE
THE CRASH-3 TRIAL

Title of Research: Tranexamic acid for the treatment of significant traumatic brain injury: an international randomised, double blind placebo controlled trial

Hospital code		Local Principal Investigator	
Patient hospital ID number		Randomisation number	BOX / PACK
Name of patient		If representative, relationship to patient	

Version Number: 1 / Version Date: 01/10/2011

1. I confirm that I have read and understood the information sheet Version Number 1, version date 01/10/2011, for the above study and have had the opportunity to ask questions.
2. I understand that my consent is voluntary and that I am free to withdraw this consent at any time, without giving any reason and without my/the patient's medical care or any legal rights being affected.
3. I understand that sections of my/the patient's medical notes may be looked at by responsible individuals involved in the study. I give permission for these individuals to have access to these records.
4. I give permission for a copy of this consent form, which contains my/the patient's personal information, to be made available to the Trial Coordinating Centre in London for monitoring purposes only.
5. I give permission for my/the patient's personal doctor to be given information about participation in this trial.
6. I agree to me/the patient taking part in the above study, the CRASH-3 trial.

______________________________	_________________	________________________________
Name of Patient/Representative	Date	Signature (thumbprint or other mark if unable to sign)

______________________________	_________________	________________________________
Name of person taking consent	Date	Signature

______________________________	_________________	________________________________
Name of site Principal Investigator	Date	Signature

The patient/representative is unable to sign. As a witness, I confirm that all the information about the trial was given and the patient/representative consented to taking part.

______________________________	_________________	________________________________
Name of witness	Date	Signature

Original to be filed in the Investigator's Study File
1 copy for patient
1 copy to be kept with patient's hospital records
